# Exportation of MDR TB to Europe from Setting with Actively Transmitted Persistent Strains in Peru

**DOI:** 10.3201/eid2503.180574

**Published:** 2019-03

**Authors:** Fermín Acosta, Juan Agapito, Andrea Maurizio Cabibbe, Tatiana Cáceres, Christophe Sola, Laura Pérez-Lago, Estefanía Abascal, Marta Herranz, Erika Meza, Bernice Klotoe, Patricia Muñoz, Gian María Rossolini, Alessandro Bartoloni, Enrico Tortoli, Daniela María Cirillo, Eduardo Gotuzzo, Darío García de Viedma

**Affiliations:** Instituto de Investigación Sanitaria Gregorio Marañón, Madrid, Spain (F. Acosta, L. Pérez-Lago, E. Abascal, M. Herranz, P. Muñoz, D. García de Viedma);; Hospital General Universitario Gregorio Marañón Servicio de Microbiología, Madrid (F. Acosta, L. Pérez-Lago, E. Abascal, M. Herranz, P. Muñoz, D. García de Viedma);; Instituto de Medicina Tropical Alexander von Humboldt, Lima, Peru (J. Agapito, T. Cáceres, E. Meza, E. Gotuzzo);; Universidad Peruana Cayetano Heredia, Lima, Peru (J. Agapito, T. Cáceres, E. Meza, E. Gotuzzo);; Istituto di Ricovero e Cura a Carattere Scientifico San Raffaele Scientific Institute, Milan, Italy (A.M. Cabibbe, E. Tortoli, D.M. Cirillo);; Institute for Integrative Biology of the Cell, CEA, CNRS, Université Paris‐Sud, Université Paris-Saclay, Gif-Sur-Yvette, France (C. Sola, B. Klotoe);; Centro de Investigación Biomédica en Red Enfermedades Respiratorias, Madrid (M. Herranz, P. Muñoz, D. García de Viedma);; Florence Careggi University Hospital, Florence, Italy (G.M. Rossolini); Careggi Hospital, Florence (A. Bartoloni)

**Keywords:** tuberculosis, *Mycobacterium tuberculosis*, MDR, migration, exportation, transmission, molecular epidemiology, Lima, Peru, Europe, Madrid, Spain, Florence, Italy, mycobacterium, MIRU-VNTR, single-nucleotide polymorphism, spoligotype, tuberculosis and other mycobacteria, antimicrobial resistance, bacteria

## Abstract

We performed a cross-border molecular epidemiology analysis of multidrug-resistant tuberculosis in Peru, Spain, and Italy. This analysis revealed frequent transmission in Peru and exportation of a strain that recreated similar levels of transmission in Europe during 2007–2017. Transnational efforts are needed to control transmission of multidrug-resistant tuberculosis globally.

International migratory movements have created a need for cross-border surveillance of tuberculosis (TB). Monitoring the transmission of multidrug-resistant (MDR) *Mycobacterium tuberculosis* strains deserves further analysis (*1*). Through migration, MDR strains can become more widely dispersed; they can be exported from the 30 countries with 89.7% of the incident MDR cases (*2*) to lower prevalence settings.

We performed a transnational molecular epidemiology analysis of MDR TB cases covering a setting with one of the highest resistance rates in Latin America (Lima, Peru) (*2*) and 2 settings in Europe hosting immigrants from Peru (Florence, Italy; and Madrid, Spain) to identify incidents of cross-border transmission. We selected 60 consecutive MDR TB cases (20% of the total MDR cases in Lima) diagnosed during 2014–2015 in one of the poorest districts of Lima (San Juan de Lurigancho), which has the highest incidence of TB (193 cases/100,000 population) in Peru (*3*). MIRU-VNTR (mycobacterial interspersed repetitive unit–variable-number tandem-repeat) analysis (Appendix 1) suggested a high percentage of recent transmission that included 36 (60%) of 60 isolates in 9 clusters (Appendix 2). A comparison of these isolates with 228 genotyped isolates from the same district 4 years earlier (*3*) revealed that 6 of the 9 strains actively transmitted during 2014–2015 were present in 2011 (Appendix 1 Table 1).

We then investigated whether some of these persistent MDR TB strains actively transmitted in Lima could have been exported to Europe. We used a dataset of 87 MIRU-VNTR genotypes of isolates in Florence obtained from TB cases in Peru during 2001–2010 (*4*) and >300 MDR genotyped isolates obtained nationwide from Italy (*5*). We found that 1 genotype matched between the Lima and Italy MDR datasets; this genotype corresponded to a strain (C8-LPMDR) that infected 11 persons in Florence and 2 in Milan during 2007–2017 (Appendix 1 Table 2). MDR TB strains from Lima were also found in Spain during 2003–2009. Three MDR isolates, matching 3 of the 9 MDR TB strains from Lima, were found in migrants from Peru residing in Madrid (Appendix 1 Table 1). One of these isolates corresponded to the active MDR strain circulating in Italy (C8-LPMDR).

We performed whole-genome sequencing (*6*) with 12 of the 17 isolates of the cross-border MDR TB cluster C8-LPMDR (7 from Florence, 2 from Milan, 2 from Lima, and 1 from Madrid). In a median-joining network analysis, these isolates were distributed along 2 branches (Figure). One branch included all the isolates from Florence. Although we lacked precise data from contact tracing to verify details regarding transmission in Florence, we were able to determine that all the Peru migrants involved came from Lima. In Florence, there is a large community of persons from Peru, which offers opportunities for interacting, such as shared residence and social gatherings. The few differences (0–2 single-nucleotide polymorphisms [SNPs]) found among these isolates strongly suggests these isolates were recently transmitted in Florence. An isolate from Lima (6068) was only 3 SNPs different from a Florence isolate, demonstrating a close genetic relationship between the Florence and Lima isolates. This close relationship also suggests that the starting point of this branch was an exportation event of an isolate from Lima. The second branch in the network includes 2 isolates identified in Europe (1 Madrid [city of origin unknown, data not available] and 1 Milan [origin Lima]) and 1 isolate identified in Lima. Because the most recent common ancestor is positioned between the 2 branches and the 2 isolates from Lima are in different branches, these branches probably represent 2 independent exportations of 2 variants of a strain prevalent in Lima that diversified after a prolonged period.

**Figure:**
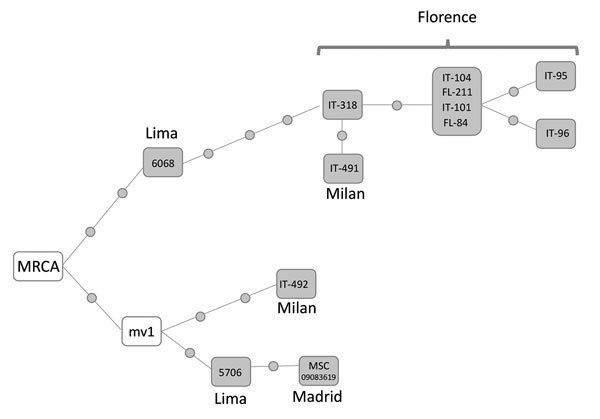
Median-joining network of whole-genome sequenced isolates of strain C8-LPMDR found in Italy, Peru, and Spain, 2007–2017. Network 4.6.1.6 (http://www.fluxus-engineering.com) was used to perform network analysis. Each dot along the lines linking isolates corresponds to a single-nucleotide polymorphism difference. Isolates within the same box share identical sequences. mv1 corresponds to an unsampled case inferred from the network topology. Sequences were deposited in the European Bioinformatics Institute database (http://www.ebi.ac.uk, accession no. PRJEB25765). FL, Florence; IT, Italy; MRCA, most recent common ancestor.

These data reveal that high-risk strains are being exported from Lima to 2 countries of Europe (Italy and Spain). Not only were these strains exported from Lima, but 1 strain caused a prolonged and ongoing transmission event in Italy. The transmission of this strain has caused at least 3 cases in Lima, 11 in Florence, 2 in Milan, and 1 in Madrid.

In another report, the international distribution of an MDR TB strain that caused 10 cases across 3 countries of Europe (Romania, Austria, and Germany) was investigated (*7*). The exportation event discussed in our report is geographically wider (intercontinental, from South America to Europe), involved more cases (17 total, with a transmission cluster of 12 cases in Italy), and occurred over a more extended period (secondary cases spanned 11 years).

Only integrative transnational efforts can provide a clearer picture of transmission of MDR TB, which has become more complex because of international migration. In this cooperative analysis involving Peru, Italy, and Spain, we detected a serious problem of active MDR TB transmission in Lima. This situation led to a pool of persistent strains that were responsible for similar transmission events after exportation to Europe via migration.

Appendix 1 Additional information on multidrug-resistant tuberculosis isolates exported from Peru to Europe.

Appendix 2 Mycobacterial interspersed repetitive unit–variable-number tandem-repeat types for multidrug-resistant isolates from San Juan de Lurigancho, Lima, 2014–2015
