# Supplementary material for: Exportation of MDR TB to Europe from Setting with Actively Transmitted Persistent Strains in Peru
Source: Emerg Infect Dis. 2019 Mar;25(3):596–8. doi: 10.3201/eid2503.180574 (PMC6390752; doi:10.3201/eid2503.180574)
Supplement: Appendix 1 — Additional information on multidrug-resistant tuberculosis isolates exported from Peru to Europe. [file 18-0574-Techapp-s1.pdf]

Article DOI: https://doi.org/10.3201/eid2503.180574

Exportation of MDR TB to Europe from Setting with Actively Transmitted Persistent Strains in Peru

Appendix 1

Actively Transmitted Multidrug-Resistant Tuberculosis Isolates in Lima, Peru

The 60 multidrug-resistant (MDR) *Mycobacterium tuberculosis* isolates from consecutively diagnosed cases occurring during 2014–2015 in San Juan de Lurigancho (district of Lima, Peru) included in this study were assigned to lineages on the basis of lineage marker single-nucleotide polymorphisms (SNPs) and/or spoligotype (*1*,*2*). Of the 60 isolates, 56 corresponded to lineage 4 (L4) and the remaining 4 to lineage 2 (L2). MIRU-VNTR (mycobacterial interspersed repetitive unit–variable-number tandem-repeat) analysis (*3*,*4*) revealed that the most frequent sublineages were Latin America–Mediterranean (L4.3, 65%), Haarlem (L4.1.2, 15%), and Beijing (L2, 6.7%) (Appendix 1 Table 3).

MIRU-VNTR–based fingerprinting indicated that 36 (60%) of 60 isolates were included in 9 clusters (C1-LPMDR to C9-LPMDR; 2–10 isolates/cluster; Appendix 2, https://wwwnc.cdc.gov/EID/article/25/3/18-0574-App2.xlsx), suggesting a high percentage of recent transmission. One of the clusters with the fewest numbers of isolates (N = 2, C9-LPMDR) was of the Beijing lineage. Each cluster included cases distributed among various health centers. The percentage of clustered cases in our analysis was higher than average values obtained elsewhere, even for susceptible isolates.

Our sample included only 20% of the total MDR isolates in Lima, and the recruitment period we used was shorter than that recommended (*5*) to efficiently capture transmission clusters. Therefore, the true proportion of transmission could be even higher. MDR *M. tuberculosis* transmission in Lima is multifactorial; in an investigation involving whole-genome sequencing and social network analysis (*6*), exposure to healthcare venues, schools, and transportation were associated with MDR *M. tuberculosis* infection.

Persistent MDR *M. tuberculosis* Strains Actively Transmitted in Lima

Once highly active transmission of MDR *M. tuberculosis* in Lima was identified, we moved on to evaluate whether the MDR *M. tuberculosis* strains that were being transmitted constituted a persistent problem in Lima. To clarify this issue, we took advantage of genotyping data available for 228 isolates (including MDR, monoresistant, polyresistant, and susceptible isolates) from new tuberculosis cases in the same district of Lima 4 years earlier (during March 2010–December 2011). The integration of both data sets revealed that 6 of the 9 clustered strains actively transmitted during 2014–2015 were previously found in 2011 (Appendix 1 Table 1). Four clusters (the 2 with the largest number of isolates [C1-LPMDR and C2-LPMDR] and 2 with few isolates [C4-LPMDR and C9-LPMDR]) of the 6 were already MDR, and 3 of these 4 strains were already part of MDR transmission clusters in 2011 (2 cases C1-LPMDR, 4 cases C9-LPMDR, and 6 cases C2-LPMDR).

References

1. Stucki D, Malla B, Hostettler S, Huna T, Feldmann J, Yeboah-Manu D, et al. Two new rapid SNP-typing methods for classifying *Mycobacterium tuberculosis* complex into the main phylogenetic lineages. PLoS One. 2012;7:e41253. PubMed http://dx.doi.org/10.1371/journal.pone.0041253

2. Molina-Moya B, Gomgnimbou MK, Lafoz C, Lacoma A, Prat C, Refrégier G, et al. Molecular characterization of *Mycobacterium tuberculosis* strains with TB-SPRINT. Am J Trop Med Hyg. 2017;97:806–9. PubMed http://dx.doi.org/10.4269/ajtmh.16-0782

3. Supply P, Allix C, Lesjean S, Cardoso-Oelemann M, Rüsch-Gerdes S, Willery E, et al. Proposal for standardization of optimized mycobacterial interspersed repetitive unit-variable-number tandem repeat typing of *Mycobacterium tuberculosis*. J Clin Microbiol. 2006;44:4498–510. PubMed http://dx.doi.org/10.1128/JCM.01392-06

4. Azé J, Sola C, Zhang J, Lafosse-Marin F, Yasmin M, Siddiqui R, et al. Genomics and machine learning for taxonomy consensus: the *Mycobacterium tuberculosis* complex paradigm. PLoS One. 2015;10:e0130912. PubMed http://dx.doi.org/10.1371/journal.pone.0130912

5. Borgdorff MW, van den Hof S, Kalisvaart N, Kremer K, van Soolingen D. Influence of sampling on clustering and associations with risk factors in the molecular epidemiology of tuberculosis. Am J Epidemiol. 2011;174:243–51. PubMed http://dx.doi.org/10.1093/aje/kwr061

6. Bui DP, Oren E, Roe DJ, Brown HE, Harris RB, Knight GM, et al. A case-control study to identify community venues associated with genetically clustered multidrug-resistant tuberculosis disease in Lima, Peru. Clin Infect Dis. 2018. PubMed http://dx.doi.org/10.1093/cid/ciy746

Appendix 1 Table 1. Isolates sharing MIRU-VNTR type with multidrug-resistant tuberculosis clusters from Lima, Peru, 2014–2015*

Cluster	No. isolates in Lima, Peru, 2014–2015	No. isolates in Lima, Peru, 2010–2011	No. isolates in Florence, Italy, 2007–2017	No. isolates in Madrid, Spain, 2003–2009	Total no. isolates
C1-LPMDR	10	3†			13
C2-LPMDR	8	20‡	2§		30
C3-LPMDR	4				4
C4-LPMDR	3	1			4
C5-LPMDR	3	1†			4
C6-LPMDR	2			1	3
C7-LPMDR	2			1	3
C8-LPMDR	2	1†	13¶	1	17
C9-LPMDR	2	4			6
Total	36				

*All numbers without a footnote symbol correspond to multidrug-resistant isolates. MIRU-VNTR, mycobacterial interspersed repetitive unit–variable-number tandem-repeat.
†One monoresistant isolate.
‡Two polyresistant, 5 monoresistant, and 7 susceptible isolates.
§One polyresistant and 1 monoresistant isolate. Any of the isolates were multidrug resistant.
¶Two of these isolates were obtained from cases in Milan, Italy.

Appendix 1 Table 2. Characteristics of isolates of cluster C8-LPMDR, Italy, 2007–2017

No.	City of isolation	Location of origin	Year of isolation
1	Florence	Latin America	2007
2	Milan	Peru	2009
3	Florence	Peru	2010
4	Milan	Latin America	2011
5	Florence	Latin America	2012
6	Florence	Peru	2013
7	Florence	Italia	2013
8	Florence	Albania	2013
9	Florence	Peru	2013
10	Florence	Latin America	2014
11	Florence	Latin America	2014
12	Florence	Peru	2016
13	Florence	Peru	2017

Appendix 1 Table 3. Multidrug-resistant isolates from Lima, Peru, 2014–2015, by lineage

Lineage	No. isolates (%)
Latin America–Mediterranean	39 (65)
Haarlem	9 (15)
Cameroon	3 (5)
X	3 (5)
S	2 (3.3)
Beijing	4 (6.7)
Total	60
